# Artificial intelligence in dementia care: challenges, controversies, and policy implications

**DOI:** 10.3389/frdem.2026.1791195

**Published:** 2026-04-10

**Authors:** Natalia Vilor-Tejedor, Robert van Vorstenbosch, Tavia E. Evans

**Affiliations:** 1 Institute for Risk Assessment Sciences (IRAS), Utrecht University, Utrecht, Netherlands; 2 BarcelonaBeta Brain Research Center, Pasqual Maragall Foundation, Barcelona, Spain; 3 Department of Human Genetics, Radboud UMC, Nijmegen, Netherlands; 4 Global Brain Health Institute, Trinity College Dublin, Dublin, Ireland

**Keywords:** artificial intelligence (AI), dementia, digital health (DH), equity and inclusion, ethics - care ethics, policy

## Abstract

Artificial intelligence (AI) is rapidly expanding into dementia-related health and social care, with proposed applications ranging from early risk detection and monitoring to care coordination and service planning. While these technologies may support independence, reduce caregiver burden, and improve efficiency in overstretched systems, dementia care is a uniquely high-stakes context for digital innovation. Cognitive decline can affect consent and agency, care often occurs in private domestic settings, and individuals may become increasingly dependent on others to interpret and act on algorithmic outputs. This Perspective examines the opportunities and challenges of AI in dementia policies and services, focusing on equity, privacy, accountability, and the risk that technologies displace human care. We argue that AI tools are only as reliable and fair as the data and infrastructures on which they depend, and that uneven access to digital resources may widen disparities in diagnosis, monitoring, and support. We also highlight often-overlooked considerations, including environmental sustainability and the broader role of AI in shaping exposures relevant to brain health across the life course. Whether AI improves dementia care will ultimately depend on policy and governance choices, including investment in equitable digital infrastructure, robust real-world validation, and safeguards that prevent technology from substituting for human care. Finally, we propose governance priorities to ensure that AI-enabled dementia innovations are implemented as a public-interest matter, grounded in meaningful engagement of people living with dementia and care partners, real-world validation, and safeguards that protect dignity, autonomy, and social legitimacy.

## Graphical abstract

**Figure:**
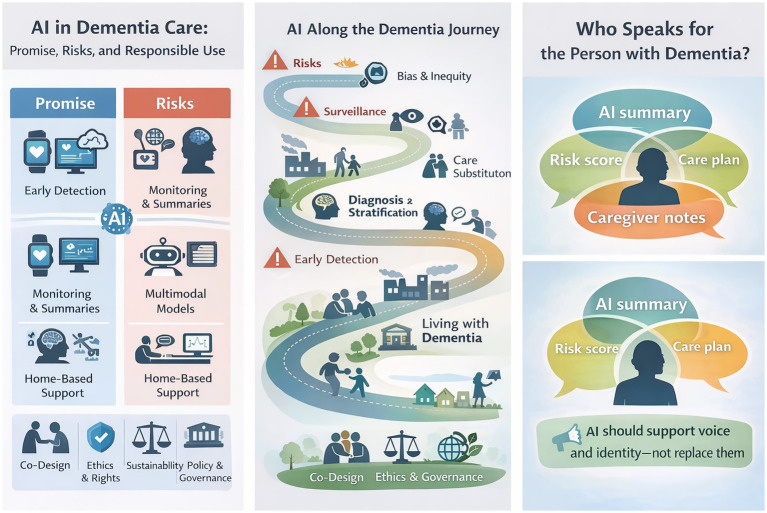
Artificial intelligence (AI) in dementia care: promise, risks, and conditions for responsible use. *Image partially generated with AI and edited in BioRender.*

## Artificial intelligence in dementia care: emerging applications

Artificial intelligence (AI) is increasingly present in daily life, from smart speakers to wearable sensors, and it is beginning to reshape how societies think about dementia-related healthcare (Wahul et al., 2025). Dementia care is now entering this same transformation, with AI-enabled tools proposed to support monitoring, care coordination, clinical decision-making, and the prediction of risk across individuals and populations (Fernandes et al., 2025).

Dementia represents one of the most costly and burdensome conditions worldwide, and health and care systems are struggling to meet rising needs (Chen et al., 2024; Nichols et al., 2022). In this context, AI is often framed to improve efficiency and extend independence by enabling earlier identification of vulnerability, reducing administrative burden, and supporting care planning in overstretched services. Yet, dementia also creates a uniquely high-stakes environment for digital health innovation: cognitive decline can compromise consent and agency, care frequently occurs within private domestic spaces, and individuals may become increasingly dependent on others to interpret and act on algorithmic outputs. As a result, the introduction of digital tools into dementia care requires stronger evidence and safeguards than in many other health contexts. The key issue is no longer whether AI will enter dementia care, but how its use will reshape care relationships, responsibilities, and access. Without appropriate governance, AI may amplify inequities, expand surveillance, and shift responsibility away from healthcare systems onto individuals and families. With the right conditions, it could support dignity, strengthen caregiver support, and improve equitable access to timely care. Framing AI as a neutral solution hides the fact that its effects will depend on who designs it, who regulates it, and which aspects of dementia care are prioritized.

AI refers to a broad family of computational methods capable of identifying complex patterns in large datasets without the need to predefine all relevant features. For example, machine learning models can detect informative patterns in ECG signals, images, language, or speech automatically, enabling the analysis of signals that may exceed human perceptual or analytical capacity. In clinical contexts, many predictive machine learning models operate within well-defined tasks (e.g., classification or risk prediction) where outputs can be externally evaluated against clinical outcomes. However, modern AI systems also include deep learning architectures and generative models such as large language models (LLMs), which produce probabilistic outputs that may appear plausible but are not grounded in explicit representations of factual truth, making them difficult to interpret, audit, or validate systematically. These differences in training objectives and evaluation paradigms raise important concerns about transparency, bias, and reliability when AI systems are deployed in real-world dementia care (Topol, 2019).

## Early detection and risk stratification

Beyond support technologies, AI is increasingly positioned to identify risk states earlier and track subtle changes over time (Tsiakiri et al., 2025). Multimodal models can integrate imaging, biomarkers, electronic health records, and high-frequency digital signals from wearables or smartphones (sleep regularity, gait proxies, speech and language features, or patterns of daily activity) (Bron et al., 2022; Vo et al., 2026). In this context, AI often functions less as a “diagnosis engine” than as a tool for estimating latent disease states (i.e., composite variables that are not directly observable but can improve risk stratification, personalize follow-up, and flag trajectories that warrant clinical review). Most of these systems produce probabilistic risk estimates rather than categorical diagnoses. Earlier detection can only be meaningful if predictive systems demonstrate reliability across real-world populations. Models trained in controlled datasets must demonstrate reliability across dementia subtypes, populations with multimorbidity, and real-world clinical environments where data are often incomplete, heterogeneous, and subject to systematic missingness. Without adequate validation, predictive models may generate misleading signals that trigger unnecessary concern or inappropriate clinical responses.

In parallel to infrastructure-intensive approaches, such as multimodal biomarker integration and continuous wearable monitoring, AI-enhanced analysis of brief cognitive assessments has emerged as a potentially scalable and lower-cost strategy for early risk detection. Machine learning models applied to speech, language, and semantic fluency tasks can detect subtle changes in lexical diversity, syntactic complexity, acoustic markers, or semantic organization that may precede clinically overt impairment. These approaches may be particularly relevant in primary care or community settings where access to advanced imaging, continuous sensing devices, or stable internet connectivity is limited. Despite their apparent accessibility, these approaches raise important methodological challenges. LMMs are highly sensitive to linguistic background, education, cultural norms, and dialect variation, raising risks of bias if training data are not sufficiently representative. Moreover, probabilistic risk outputs derived from brief tasks must be interpreted cautiously to avoid overmedicalization of normative cognitive variability. As with other AI systems, validation across diverse populations is essential.

In clinical and community care, AI could also translate complex information into interpretable summaries for patients, carers, and clinicians, for example, detecting patterns of nocturnal wandering, agitation episodes, or medication nonadherence from passive sensing, as well as sleep alterations in caregivers (Palmese et al., 2025). Such summaries could support care planning and reduce documentation burden, but they may also introduce new forms of surveillance and generate false alarms, anxiety, or overreliance on algorithmic outputs as a substitute for clinical judgement. Recent evaluations of commercial generative AI systems in clinical triage contexts have highlighted substantial variability in recommendations and potential safety risks when outputs are interpreted without expert oversight (Ramaswamy et al., 2026).

AI also interacts with dementia across the lifespan, not only through care technologies but through its role in shaping digital environments and quantifying exposures at scale (Lyall et al., 2023). This aligns with the exposome framework, which emphasizes that lifelong environmental and social determinants contribute to brain aging and dementia risk (Lansbury, 2024; The Human Exposome Project, 2026). Digital systems can influence diet, physical activity, stress, sleep, mobility, and social connection, while AI can help integrate exposome indicators (pollution, noise, green space, socioeconomic context) with genetics and biomarker profiles to understand risk trajectories. However, these systems may also reinforce harmful exposures through targeted advertising, algorithmic stressors, or unequal access to healthy environments. In this sense, AI has a dual role within the exposome framework. First, AI methods can help integrate heterogeneous environmental and social data (e.g., pollution exposure, neighborhood characteristics, mobility patterns) with biological and clinical information to better understand dementia risk trajectories. Second, digital technologies increasingly influence daily exposures, including patterns of activity, social interaction, stress, and information access. Considering AI within the exposome framework highlights both its analytical role and its influence on environmental determinants of brain health. In this sense, AI becomes relevant to dementia risk both as an analytical tool used to study environmental exposures and as a technological system that may itself shape behavioral and environmental determinants of brain health. Without equitable access, AI-enabled prevention strategies risk reinforcing existing social inequalities, and potential harms should be addressed through enforceable governance and regulation.

## Equity, representation, and digital exclusion

Despite their potential, dementia-focused AI tools must be designed and validated for safe and equitable use in real-world populations, as not all systems will be equally accessible, reliable, or clinically appropriate. AI methods are only as robust as the data on which they are trained; models developed in selective or unrepresentative datasets may perform poorly when applied to different clinical or demographic groups. This challenge is particularly relevant in dementia care because “dementia” is not a single diagnosis but an umbrella term covering multiple etiologies and syndromes with highly variable trajectories, symptoms, and care needs. Consequently, the effectiveness and acceptability of AI will differ across disease stage, clinical presentation, living situation, socioeconomic context, language, and cultural background (Chu et al., 2022).

For example, fall-detection algorithms calibrated on younger or healthier bodies may misclassify movement patterns in frail older adults - an issue relevant to ageing more broadly- but evidence for robust performance in dementia populations remains limited, despite the high stakes of misclassification in dependent care settings (Eost-Telling et al., 2024). More generally, AI tools should avoid “one-size-fits-all” approaches and instead target specific symptoms, settings, or decision points, particularly given the heterogeneity of dementia syndromes. Predictive models may also overlook cultural and regional differences, including variation in caregiving norms, service use, and healthcare pathways that shape real-world care demand (Tran et al., 2025). While the inclusion of diverse datasets is a basic requirement for safe and generalizable AI, this condition is rarely met in practice, and tools may therefore be deployed beyond the contexts for which they were adequately validated.

Beyond representativeness and model validity, the benefits of AI may also be unequally distributed. Many AI tools depend on smartphones, reliable internet connectivity, digital literacy, or stable housing conditions, which can systematically exclude groups already facing barriers to dementia care. This digital exclusion can reinforce gaps in training data and deployment, leading to persistent underrepresentation of racialized communities, individuals with lower socioeconomic status, and people from diverse linguistic or cultural backgrounds. If not addressed, these structural limitations may contribute to poorer performance and reduced benefit in precisely the populations where unmet need is often greatest, widening disparities in dementia diagnosis, monitoring, and support (Duan et al., 2024; Veinot et al., 2018).

## Environmental sustainability and structural impacts

Equity considerations also extend beyond clinical effectiveness and access, to include the broader societal and environmental costs of AI deployment. AI technologies are frequently justified as tools to improve efficiency and reduce the growing economic burden of dementia on health and care systems. However, training and operating large-scale AI systems can require substantial computational resources, with associated energy demand, water use for cooling, and expansion of digital infrastructure (Atianashie et al., 2025; Artificial Intelligence: How Much Energy Does AI Use? - United Nations Western Europe, 2025). These environmental costs raise questions about whether efficiency gains in healthcare may be partially offset by wider societal externalities, particularly given growing evidence linking environmental exposures such as air pollution and climate-related stressors to cognitive decline and dementia risk. These costs are not evenly distributed, due to data centers and energy-intensive computing facilities may concentrate near specific communities, raising concerns about environmental justice and local resource use. In addition, long-term dependence on commercial AI providers may create structural vulnerabilities for health systems, particularly if expertise within public institutions is reduced or systems become locked into proprietary platforms. A dementia-focused AI agenda that prioritizes dignity and equity should therefore incorporate environmental sustainability, long-term affordability, and institutional resilience into procurement and regulatory decisions (Roadmap Shows the Environmental Impact of AI Data Center Boom | Cornell Chronicle, 2026).

## Privacy, surveillance, and autonomy

Another core concern is privacy. Dementia-related AI often relies on continuous data collection in private spaces, including homes, care facilities, or on the body (Hartmann et al., 2022). Monitoring technologies can shift from safety support to intrusive surveillance, especially when a person with dementia cannot fully consent or meaningfully withdraw (Hall et al., 2019). The ethical dilemma becomes clear: while families and clinicians may welcome technologies that promote safety, individuals with dementia may experience these tools as surveillance, and data sharing between families, clinicians, insurers, or commercial platforms may occur without transparent oversight. Balancing protection with respect for autonomy requires ongoing negotiation of acceptable trade-offs, safeguards that adapt to changing capacity over time, and clear accountability for data governance. Patient experience should be the foundation of implementation, and privacy could not be treated as an individual preference alone but as a structural condition for dignity and autonomy (Ballegaard et al., 2025; Ploug et al., 2025).

## Substitution versus complementarity in care

A further risk is that AI-enabled technologies are treated as substitutes for human care rather than complements to it. Social robots and conversational agents may support routines, reminders, or perceived companionship, but their adoption could also legitimize reduced staffing or shift caregiving responsibilities from families and services onto devices (Łukasik and Gut, 2025). Even when experienced as helpful, such tools may inadvertently deepen loneliness if they displace, rather than augment, human relationships. Dementia care depends on trust, empathy, and relational continuity, qualities that current AI cannot reproduce (Kerasidou, 2020). AI should therefore not be pursued primarily as a route to efficiency, but as a tool that supports more humane care. If AI is implemented mainly as a cost-saving strategy without parallel investment in the care workforce and community-based services, the outcome may be an “efficient” yet impoverished model that meets physical needs while neglecting emotional and social dimensions. Trust is also not uniform; it is shaped by local histories, institutional credibility, and cultural expectations about privacy and technology. Governance must therefore protect social legitimacy as well as technical performance, through transparency, accountability, and meaningful community oversight.

AI may also affect how people with dementia are represented and heard. Tools that generate summaries of behavior, predict needs, or suggest interventions can inadvertently “speak for” the person, reducing their identity to patterns or risk scores. At the same time, carefully designed AI may support communication, for example, simplifying medical information, helping people express preference, or supporting reminiscence through personalized prompts (Astell et al., 2019; Moyle et al., 2017). Some proposals involve “digital memory” systems that preserve biographical details, relationships, or routines to help patients and families maintain continuity of identity. These applications may be valuable, but only if governance is clear: who curates the content, how consent is maintained as capacity changes, and how errors or misrepresentations are corrected.

## Governance, co-production, and public-interest AI

Despite these concerns, the goal should not be to resist AI, but to shape its development and deployment responsibly. There are important opportunities to integrate AI into dementia services in ways that uphold dignity, equity, and inclusion, and a key step is to move beyond technology-first approaches (Agbana, 2025). People living with dementia and carers must be involved not only as “users” but as co-designers and decision-makers.

Emerging implementation research underscores that co-production is not only a normative ethical aspiration but a practical requirement for safe deployment (Astobiza et al., 2025). Stakeholder-informed studies of AI-supported dementia screening and monitoring have identified concerns that were not fully anticipated during technical development, including anxiety triggered by false-positive alerts, distrust of automated triage systems, uncertainty regarding clinical responsibility, and misalignment with existing workflows (Moldt et al., 2025). In some contexts, clinicians report apprehension about liability and the interpretability of model outputs, while patients and families express ambivalence about continuous monitoring despite acknowledging safety benefits (Maliha et al., 2021). These findings demonstrate that technical performance alone does not determine real-world success; legitimacy, trust, and workflow integration are equally decisive (Astobiza et al., 2025). Embedding stakeholder engagement early in design and validation processes strengthens both ethical robustness and implementation feasibility.

Co-production can help determine which outcomes matter most (e.g., reduced caregiver stress or preserved autonomy rather than “engagement time”), define acceptable trade-offs between safety and privacy (e.g., on-device processing rather than cloud upload, or “check-in” prompts rather than continuous monitoring), and ensure escalation pathways to a human professional when distress or confusion is detected. It can also surface practical barriers early, such as stigma, low digital confidence, or multi-user household dynamics, so that tools are designed for real-world adoption rather than idealized settings.

Collaboration across sectors will be essential. Governments, researchers, technology companies, healthcare services and civil society organizations each bring distinct expertise and priorities, and shared accountability is more likely to produce systems that are both effective and trustworthy. Alongside this, there is a strong case for dementia-specific ethical frameworks for AI, including requirements for validation in real-world settings, monitoring for algorithmic drift over time, and safeguards to ensure that commercial incentives do not override patient welfare. From a policy perspective, responsible integration of AI into dementia care requires concrete governance mechanisms. These include mandatory real-world validation before clinical deployment, transparency requirements regarding training data and model limitations, procurement policies that avoid dependence on proprietary platforms, and regulatory oversight that ensures AI tools complement rather than replace human care provision.

## Geopolitics, regulation, and system dependence

A broader challenge is that AI is developing within a rapidly changing geopolitical landscape. As AI technologies become strategically valuable, competition between states and corporations may influence which tools are prioritized, how data is governed, and what ethical standards are enforced. Some jurisdictions are strengthening regulations for medical AI, while others encourage rapid deployment (van Kolfschooten and van Oirschot, 2024). Dementia care systems may therefore become dependent on external technology providers, raising concerns about sovereignty, resilience, and long-term affordability. These pressures reinforce the importance of explicit dementia policy positions on AI procurement, public–private partnerships, and the ownership of data infrastructures.

In practice, the effects of AI on dementia care will depend on the governance frameworks that shape its deployment. If AI tools are introduced primarily to cut costs, expand surveillance, or shift responsibility away from services, they may erode autonomy and deepen inequities. If they are designed and governed to support agencies, strengthen relationships, and reduce structural barriers to healthy ageing, they may contribute to more humane and sustainable care systems (Fan, 2025). We therefore argue for a policy approach that treats AI in dementia as a public-interest matter (WHO, 2021). This requires investment in equitable digital infrastructure, enforceable standards for privacy and accountability, and mechanisms for meaningful participation by people affected by dementia. It also requires resisting the framing of AI as a neutral “solution” and instead recognizing it as a set of choices about what societies value in care.

## Conclusion: conditions for responsible integration

Any benefit from AI in dementia care will depend less on the technology itself than on the governance decisions surrounding its validation, access, and use. The goal should not be to replace caregivers or outsource responsibility to algorithms, but to shape AI as a tool that strengthens dignity, autonomy, and wellbeing throughout the dementia journey.

## Data Availability

Publicly available datasets were analyzed in this study. This data can be found at: no data used.
